# Diversity of malaria parasites in great apes in Gabon

**DOI:** 10.1186/s12936-015-0622-6

**Published:** 2015-03-14

**Authors:** Larson Boundenga, Benjamin Ollomo, Virginie Rougeron, Lauriane Yacka Mouele, Bertrand Mve-Ondo, Lucrèce M Delicat-Loembet, Nancy Diamella Moukodoum, Alain Prince Okouga, Céline Arnathau, Eric Elguero, Patrick Durand, Florian Liégeois, Vanina Boué, Peggy Motsch, Guillaume Le Flohic, Alphonse Ndoungouet, Christophe Paupy, Cheikh Tidiane Ba, Francois Renaud, Franck Prugnolle

**Affiliations:** Centre International de Recherche Médicale de Franceville, BP 769, Franceville-Gabon, Gabon; MIVEGEC (Laboratoire Maladies Infectieuses et Vecteurs, Ecologie, Génétique, Evolution et Contrôle), UMR CNRS 5290 / IRD 224, Université Montpellier 1, Montpellier, France; Laboratoire d’Écologie et Biologie évolutive, Département de Biologie Animale, Faculté des Sciences et Techniques, Université Cheikh Anta Diop de Dakar, Dakar, BP5005 Sénégal; TransVIHMI (Recherche Translationnelle sur le VIH et les Maladies Infectieuses), UMI 233, Institut de Recherche pour le Développement (IRD) and Université Montpellier 1, Montpellier, France

**Keywords:** Plasmodial diversity, *Laverania* clade, Great apes, *Cytochrome-b*, Gabon

## Abstract

**Background:**

Until 2009, the *Laverania* subgenus counted only two representatives: *Plasmodium falciparum* and *Plasmodium reichenowi*. The recent development of non-invasive methods allowed re-exploration of plasmodial diversity in African apes. Although a large number of great ape populations have now been studied regarding *Plasmodium* infections in Africa, there are still vast areas of their distribution that remained unexplored. Gabon constitutes an important part of the range of western central African great ape subspecies *(Pan troglodytes troglodytes* and *Gorilla gorilla gorilla*), but has not been studied so far. In the present study, the diversity of *Plasmodium* species circulating in great apes in Gabon was analysed.

**Methods:**

The analysis of 1,261 faecal samples from 791 chimpanzees and 470 gorillas collected from 24 sites all over Gabon was performed. *Plasmodium* infections were characterized by amplification and sequencing of a portion of the *Plasmodium cytochrome b* gene.

**Results:**

The analysis of the 1,261 samples revealed that at least six *Plasmodium* species circulate in great apes in Gabon (*Plasmodium praefalciparum, Plasmodium gorA (syn Plasmodium adleri), Plasmodium gorB (syn Plasmodium blacklocki)* in gorillas and *Plasmodium gaboni, P. reichenowi* and *Plasmodium billcollinsi* in chimpanzees). No new phylogenetic lineages were discovered. The average infection rate was 21.3% for gorillas and 15.4% for chimpanzees. A logistic regression showed that the probability of infection was significantly dependent on the freshness of the droppings but not of the host species or of the average pluviometry of the months of collection.

## Background

*Plasmodium falciparum* is a protozoan parasite responsible for malaria in humans. Among the five parasites infecting humans (*Plasmodium falciparum, Plasmodium malariae, Plasmodium ovale, Plasmodium vivax* and *Plasmodium knowlesi*), *P. falciparum* is by far the most virulent, responsible every year for approximately 207 million clinical cases and 627,000 deaths in the world [1], of which 98% are in sub-Saharan Africa [2-5]. Malaria is proving to be an obstacle that can slow down economic prosperity in many tropical countries, particularly in Africa [3].

*Plasmodium falciparum* belongs to the subgenus *Laverania,* which up to 2009 included only two known representatives: *P. falciparum* and *Plasmodium reichenowi*, a parasite from chimpanzees. Since 2009, thanks to the use of molecular tools for species identification and the development of non-invasive methods, several studies re-explored the diversity of *Plasmodium* species circulating in non-human primates in Africa, especially great apes (gorillas and chimpanzees) [6-8]. These studies revealed the existence of several lineages/species related to *P. falciparum*, deeply modifying the comprehension of the evolution of this parasite and of *Laverania* more generally. Four *Laverania* species are now recognized to infect chimpanzees: *P. reichenowi, Plasmodium billcollinsi, Plasmodium gaboni* and *Plasmodium billbrayi* [9-11]. For gorillas, there are three species: *Plasmodium praefalciparum* (the closest relative of *P. falciparum*), *Plasmodium gorB* (syn-*Plasmodium blacklocki*) and *Plasmodium gorA* (syn-*Plasmodium adleri*) [2,12]. Great apes have also been shown to be infected with species of the subgenus *Plasmodium* (non*-Laverania*): *P. malariae*-like, *P. ovale*-like and *P. vivax*-like parasites [8,13,14].

Was the entire diversity of *Plasmodium* species circulating in great apes in Africa discovered? Although a large number of great ape populations in Africa have now been studied regarding *Plasmodium* infections, there are still vast areas of their geographic distribution that remain unexplored. This is the case, for instance, for the western, central African populations of chimpanzees and gorillas (*Pan troglodytes troglodytes* and *Gorilla gorilla gorilla*). Although the range of both species covers all Gabon, half the surface of the Republic of the Congo, the south of Cameroon (south of the Sanaga river) and south of the Central African Republic, *Plasmodium* infections were almost only studied in populations from Cameroon, making about two-thirds of their range still unexplored [2,3,12].

In this study, using the second largest bank of faecal samples studied so far (more than 1,200 faecal samples), the diversity of *Plasmodium* species circulating in the great ape populations of Gabon was analysed. An investigation of the ecological factors susceptible to influence the detection of *Plasmodium* from these non-invasive samples was also performed.

## Methods

### Origin of faecal samples

**Figure 1:**
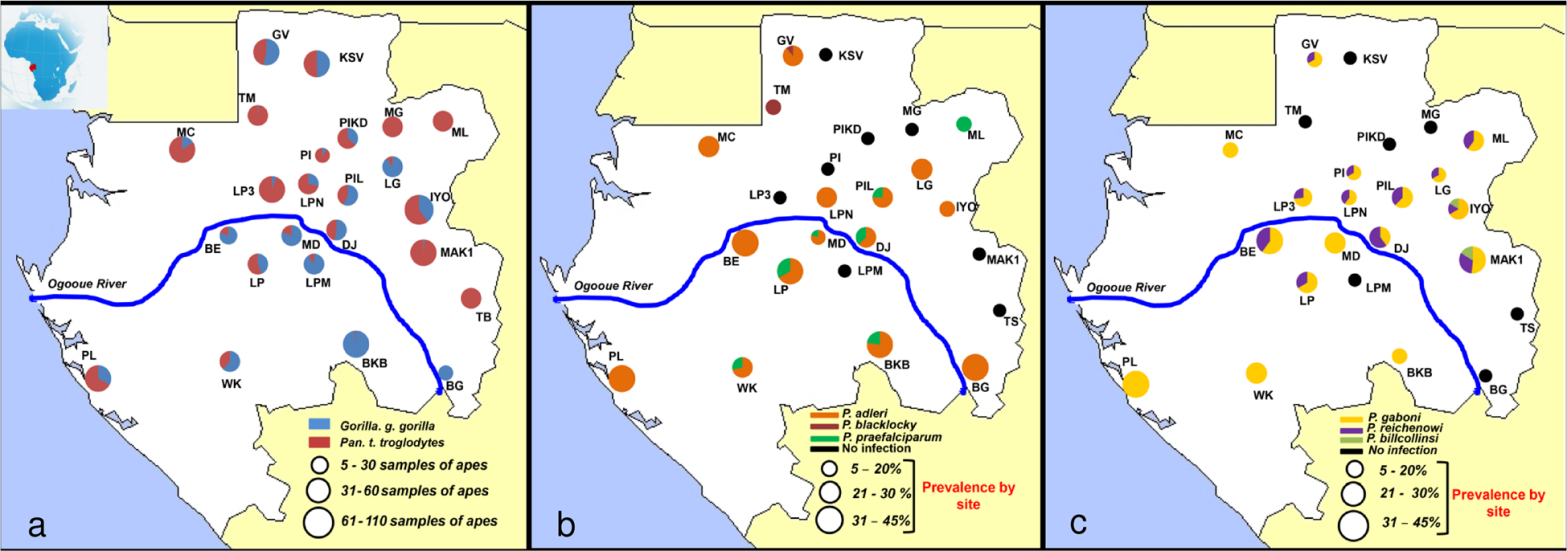
**Sampling sites and variations of** ***Plasmodium*** **prevalences in Gabon. (a)** Distribution of the sampling sites and amount of gorilla and chimpanzee samples collected and analysed in each site. Figure also shows the variations of prevalence (frequency of PCR-positives) and relative frequencies among positives of the different parasite species within the various populations of gorillas **(b)** and chimpanzees **(c)** sampled.

Faecal samples of chimpanzees and gorillas were collected in 24 sites in Gabon from 2010 to 2014 (Figure 1a and Table 1). In the field, the origin of the faeces (chimpanzee or gorilla) was deduced according to cues such as the type of nest near which they were found, footprints, texture, and odours. Freshness of the faeces (>24or <24 hours post excretion) was also estimated based on the freshness of surrounding nests (when present), texture, colours, humidity, and level of degradation. All samples were preserved in RNA*later*® (Life technologies, USA) and conserved at the CIRMF at -80°C. Their origin (chimpanzee or gorilla) was confirmed by mitochondrial DNA analysis as previously described [15,16]. This investigation was approved by the Government of the Republic of Gabon and with the authorization of the Agence Nationale des Parcs Nationaux (ANPN). In total, 791 faecal samples of chimpanzees and 470 of gorillas were collected and analysed.

**Table 1:** **List of collection sites, abbreviations and geographical coordinates in Gabon**

**Sites**	**Abbreviations**	**Coordinates**	
		**(Degree, minute, second)**	
Lope	**LP**	**S0°13′21.5″**	**E11°36′ 37.5″**
Lope-Mikongo	**LPM**	**S0°18′27.2″**	**E11°40′10.3″**
Tsouba	**TB**	**S1°09′54.2″**	**E14°26′46.8″**
Makande	**MD**	**S0°40′53.7″**	**E11°55′34.4″**
Langoue	**LG**	**N0°00′05.8″**	**E12°27′25.9″**
Parc Ivindo-Langoue	**PIL**	**S0°11′23.0″**	**E12°34′58.4″**
Parc Ivindo-Iret-Kongou-Djidji	**PIKD**	**N0°30′05.2″**	**E12°48′ 03.4″**
Makatamangoye 1	**MAK1**	**S0°08′39.3″**	**E13°36′47.6″**
Monts De Cristal	**MC**	**N0°40′15.4″**	**E10°24′54.2″**
Parc Ivindo	**PI**	**N0°23′24.8″**	**E12°41′33.1″**
Djidji	**DJ**	**N0°10′41.2″**	**E12°43′51.8″**
Mwagna	**MG**	**N0°38′53.5″**	**E13°52′08.2″**
Boumango	**BG**	**S1°43′36.0″**	**E14°03′10.0″**
Malouma	**ML**	**N0°39′01.6″**	**E13°52′17.2″**
Lope 3	**LP3**	**S0°19′32.4″**	**E11°37′23.6″**
Gabonville	**GV**	**N1°46′55.7″**	**E11°56′58.4″**
Tomassi	**TM**	**N1° 06′37.0″**	**E11°42′42.4″**
Iyokomilieu	**IYO**	**N0°02′54.1″**	**E13°36′05.6″**
Boue	**BE**	**S0°11′52. 7″**	**E12°02′01.8″**
Parc de Loango	**PL**	**S1°59′54.8″**	**E9°27′10.5″**
Waka	**WK**	**S1°07′57.3″**	**E11°08′30.8″**
Bakoumba	**BKB**	**S1°45′47.8″**	**E12°57′06.2″**
Lope-Nord	**LPN**	**N0°18′52.1″**	**E12°34′37.7″**
Konossaville	**KSV**	**N1°40′23.9″**	**E12°04′ 09.7″**

### Extraction of DNA and PCR

Faecal DNA was extracted using the QIAamp DNA Stool Mini Kit (Qiagen, Courteboeuf, France) as previously described [17] and *Plasmodium* infections were determined after amplification of a portion of *Plasmodium* mitochondrial genome (*cytochrome b*: *cyt-b*) as described in Prugnolle *et al.* [2]. All amplified products (10 μl) were run on 1.5% agarose gels in TAE buffer. The PCR-amplified products (956 bp) were used as templates for sequencing. DNA sequencing was performed by Eurofin MWG [18].

### Species identification in mixed infections

When sequence chromatograms showed multiple peaks (heterozygous base calling), the program Mixed Sequences Reader (MSR) was used to determine if the isolates were mixed infected and by which species [19]. This program can directly analyse heterozygous base-calling fluorescence chromatograms and identify species in presence from a list of reference sequences (Table 2).

**Table 2:** **Percentage of mixed infections detected from sequence chromatograms with multiple peaks using the program MSR (Mixed Sequences Reader)**

**Host**	**Percentage (%) of mixed infections**	**Associated species (n)**
		*P. reichenowi + P. gaboni*(6)
		*P. billcollinsi + P. gaboni*(1)
Chimpanzees	20% (8/40)	*P. reichenowi + P. billcollinsi*(1)
Gorillas	28% (8/29)	*P. adleri + P. praefalciparum*(7)
		*P. adleri + P. blacklocky*(1)

n: Number of mixed infection found.

### Phylogenetic analyses

Phylogenetic analyses were performed using only *cyt-b* sequences derived from chromatograms with no ambiguous base calls. To examine the relationship of the *cyt-b* sequences obtained with the different *Plasmodium* species known so far, a phylogenetic tree was constructed using a set of reference sequences belonging to different *Plasmodium* species. Hosts and GenBank accession numbers for these reference sequences are given in Table 3. The multiple alignment of all partial *cyt-b* sequences (686 nucleotides) was done using ClustalW (v 1.8.1 in BioEdit v.7.0.9.0. software) [20]. Maximum likelihood (ML) tree construction was based on the *cyt-b* sequences. The best-fitting ML model under the Akaike Information Criterion was GTR (general time reversible) + ModelTest. [21] The highest-likelihood DNA tree and corresponding bootstrap support values were obtained by PhyML (freely available at the ATGC bioinformatics platform [22,23]) using NNI (nearest neighbour interchange) + SPR (sub-tree pruning regrafting) branch swapping and 100 bootstrap replicates [24].

**Figure 2:**
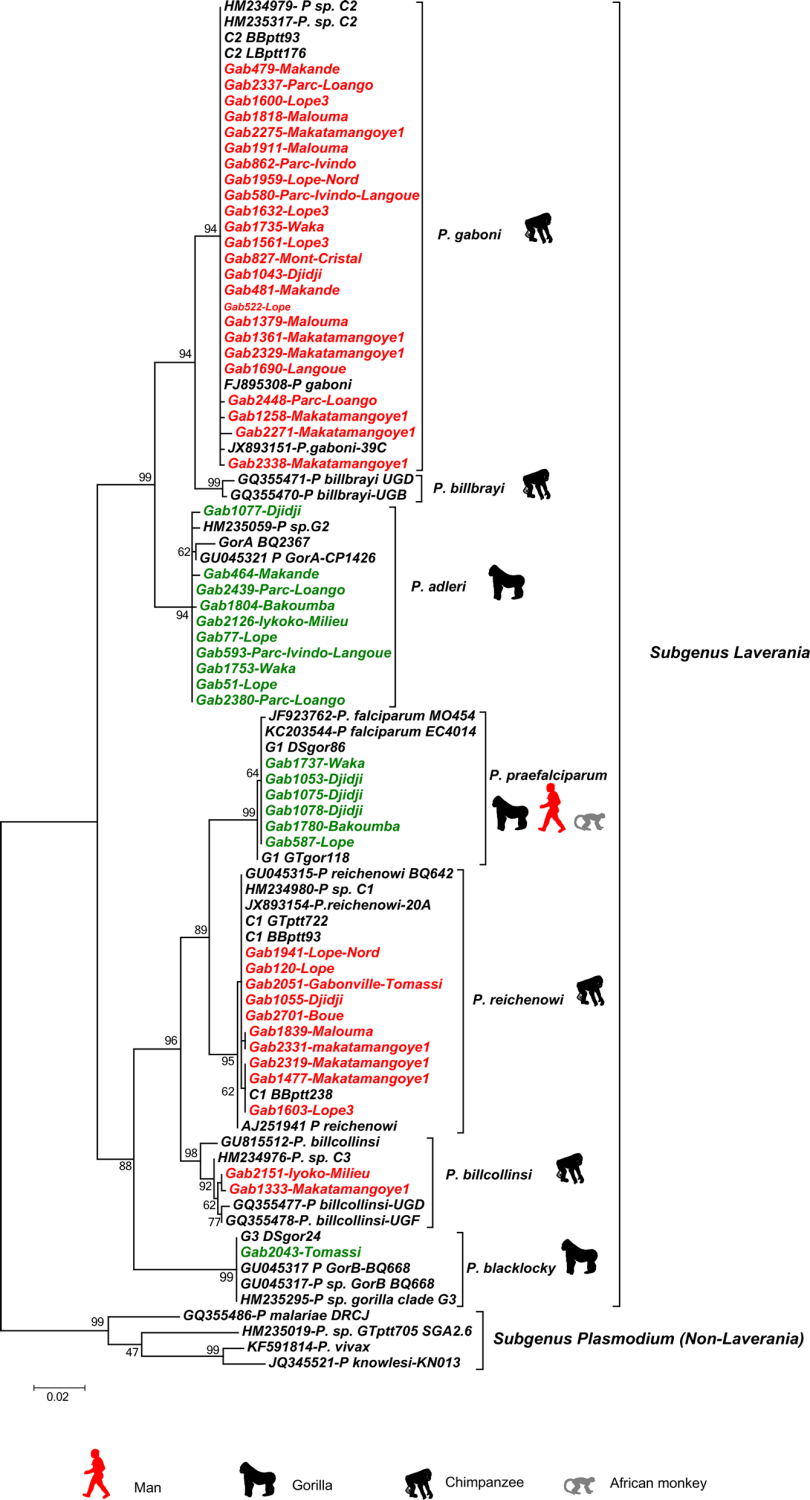
**Phylogenetic relationships between the** ***Cytochrome b*** **sequences obtained in the study and those of known** ***Plasmodium*** **species (represented by their accession number).** The tree was built based on *cytochrome b* (*cyt-b*) sequences of 686 bp. Red indicates sequences obtained from chimpanzees and green from gorillas. Bootstrap values are given at each node. More details on the different reference sequences can be found in Table 3.

**Table 3:** **Accession numbers of the sequences of reference used in the phylogenetic tree**

**Accession number**	**Isolates**	**Species**	**Host species**	**References**
**HM235178**	C1 BBptt238	*P. reichenowi*	Chimpanzee	Liu *et al.* [12]
**HM235317**	C2 LBptt176	*P. gaboni*	Chimpanzee	Liu *et al.* [12]
**HM234979**	C2 BBptt93	*P. gaboni*	Chimpanzee	Liu *et al.* [12]
**HM234980**	C1 BBptt93	*P. reichenowi*	Chimpanzee	Liu *et al.* [12]
**HM234976**	C3 BApts1413	*P. billcollinsi*	Chimpanzee	Liu *et al.* [12]
**KC203544**	EC4014_SGA500.11	*P. falciparum*	Human	Sundararaman *et al.* [25]
**FJ895308**	Isolate B	*P. gaboni*	Chimpanzee	Ollomo *et al*. [11]
**GU045315**	BQ642	*P. reichenowi*	Chimpanzee	Prugnolle *et al*. [2]
**GQ355486**	DRCJ	*P. malariae*	Bonobo	Krief *et al*. [26]
**FJ409564**	CPZcam91	*P. ovale*	Chimpanzee	Duval *et al*. [27]
**KF591814**	MRL49_FD_SGA1k.	*P. vivax*	Human	Liu *et al.* [14]
**JQ345521**	KN013	*P. knowlesi*	Human	Neoh Wan Fen *et al*. [28]
**GU045317**	BQ668	*P. blacklocki*	Gorilla	Prugnolle *et al*. [2]
**GU045322**	BQ638	*P. adleri*	Gorilla	Prugnolle *et al*. [2]
**HM235386**	G1 DDgor27	*P. praefalciparum*	Gorilla	Liu *et al.* [12]
**HM235295**	G3 DSgor24	*P. blacklocki*	Gorilla	Liu *et al.* [12]
**HM235203**	G1 DSgor86	*P. praefalciparum*	Gorilla	Liu *et al.* [12]
**HM235059**	G2 KKgor2638	*P. adleri*	Gorilla	Liu *et al.* [12]
**JF923762**	MO454	*P. praefalciparum*	*C. nictitans*	Prugnolle *et al*. [2]
**GU815512**	Louise	*P. billcollinsi*	Chimpanzee	Kaiser *et al.* [8]
**GQ355478**	UGF	*P. billcollinsi*	Chimpanzee	Krief *et al*. [26]
**GQ355477**	UGD	*P. billcollinsi*	Chimpanzee	Krief *et al*. [26]
**AJ251941**	-	*P. reichenowi*	Chimpanzee	Conway *et al*. [29]
**JX893151**	Clone39C	*P. gaboni*	Chimpanzee	Pacheco *et al*. [30]
**JX893154**	Clone20A	*P. reichnowi*	Chimpanzee	Pacheco *et al.* [30]

### Statistical analyses

All statistical analyses were performed using R [31]. A logistic regression was used to analyse the variations among individuals in the infection status. In these models, the variable to be predicted was the presence/absence of a *Plasmodium* infection. The predictive variables were: (i) the site of collection (random effect); (ii) freshness of the faeces; (iii) host species; and, (iv) average pluviometry during months of collection (fixed effects). For the second predictive variable, faeces were subdivided into two groups: the faecal samples deposited less than 24 hours before collection and those collected after 24 hours. The host species corresponded to gorilla and chimpanzee. Finally, for each month of collection, the average Gabonese pluviometry (estimated from data collected from1960 to 1990) was retrieved from [32], which data were produced by the Climatic Research Unit (CRU) of University of East Anglia (UEA). Pluviometry was considered as a possible predictive variable because it is known to influence levels of infection in human foci [33,34].

### GeneBank accession numbers published in this study

The sequences reported in this study were deposited in GenBank under the following accession numbers KP875428 to KP875480

## Results

### *Plasmodium* species infecting great apes in Gabon

Some 1,261 faecal samples from wild chimpanzees (n = 791) and gorillas (n = 470) from 24 sites were analysed (Figure 1a). Among them, 122 samples of chimpanzees (15.42%) and 100 of gorillas (21.28%) were detected positive to a *Plasmodium* infection by *Cyt-b* PCR. Sequences of quality (of sufficient size (>600 bp) and with a clear chromatogram) were obtained for 31% (n = 69) of the *Cyt-b* amplicons. Among them, sixteen showed multiple peaks and were identify as clear mixed infections by the program MSR. The frequency of mixed infections observed in chimpanzees and gorillas as estimated by the analysis of the chromatograms is given in Table 2. Phylogenetic analyses (Figure 2) revealed the presence of three *Plasmodium* species in chimpanzees (*P. gaboni, P. reichenowi* and *P. billcollinsi*) and three in gorillas (*P. praefalciparum, P. gorA* and *P. gorB*). Neither species of the subgenus *Plasmodium* (*P. vivax-*like*, P. malariae-*like and *P. ovale-*like) nor new phylogenetic lineages were found in these samples. Relative frequencies of each *Plasmodium* species in each site among positives are given in Figure 1a-c.

Over the entire dataset, logistic regressions revealed that the probability of infection was only significantly dependent on the variable ‘freshness of the stool’. Pluviometry as well as host species did not significantly explain the probability of infection (Table 4). As shown in Table 4, the probability of infection was higher in stools collected less than 24 hours after dropping than in the older ones. Overall, freshness of the stools did not significantly differ between chimpanzees and gorillas (*p-value* = 0.07).

**Table 4:** **Results of the logistic regression**

**Variable**	***P-value***	**Odds ratio [CI**_**95%**_ **]**
Host species	0.051390	0.67 [0.503 to 0. 905]
Freshness of the faeces	0.006684	2.038[1.458 to 2. 849]
Pluviometry	0.581011	0.576 [0.429 to 0. 775]

The presence or absence of infection by *Plasmodium* was the variable to be predicted*.* Predictive variables were: host species, freshness of faeces (<24 or >24 hr) and pluviometry. CI_95%_: 95% Confidence Interval.

## Discussion

In the last few years, several new *Plasmodium* species were discovered in African non-human primates, especially great apes [2,3,6,10,26]. These discoveries were made possible by the development of a non-invasive method allowing detection of *Plasmodium* infections from faecal samples [2,6], despite inherent problems of DNA degradation with this type of biological material. This issue was overcome by the use of mitochondrial sequences to amplify the parasite, which presents several advantages: 1) Mitochondrial DNA is in multiple copy inside parasites (unlike nuclear DNA) and 2) if properly chosen, small portions of the mitochondrial genome (as small as 200 bp), can contain enough phylogenetic information to identify the different *Plasmodium* species. This method is now one of the main methods used to analyse *Plasmodium* from wild non-human primates [2,7,12,25].

In the present study, analyses were performed on a set of 1,261 faecal samples collected all over Gabon from chimpanzees and gorillas. All *Plasmodium* species found belonged to the subgenus *Laverania* and were all previously identified in *Pan troglodytes troglodytes* and *Gorilla gorilla gorilla*, respectively [2,12]. No new phylogenetic lineage or species were identified. Surprisingly, no species of the subgenus *Plasmodium* (*non-Laverania*) were identified either. This is at odds with recent observations made from ape blood samples or infected sylvatic anopheline mosquitoes collected in Gabon showing the circulation of *P. vivax-*like parasites in the area. [13] These results are nevertheless congruent with those from Liu *et al*. [12]. Although they analysed 3,000 ape faecal samples from west and central Africa, they only obtained seven sequences of *Plasmodium* belonging to the subgenus *Plasmodium* (non*-Laverania*).

One main factor could explain why parasites of the subgenus *Plasmodium* were not detected and this is most likely linked to the nature of the primers used to perform PCRs. Indeed, as in the study of Liu *et al.* [12], the primers used were specifically designed to amplify sequences of *Laverania* parasites. As a consequence, several nucleotides of differences separated them from the homologous sequences in *P. vivax, P. malariae* and *P. ovale*, thus very likely reducing the sensitivity of this PCR to detect non*-Laverania* species. In addition, such problem might have been amplified by the presence of co-infections with *Laverania* species. Indeed, it has been demonstrated that in case of co-infection, the PCR tends to favour the amplification of the parasite with the best matching sequence to the primers [35,36]. Recently, Liu *et al*. [14] solved this problem by designing primers specific to *P. vivax*. Out of the 3,000 samples previously analysed and re-analysed with other samples, they finally detected more than 87 *P. vivax* infections.

In this study, no *Laverania* species were found to infect both hosts (gorillas and chimpanzees). This reinforces the hypothesis that *Laverania* lineages infect specific hosts [10], a specificity that could be associated to specific ligand/receptor interactions occurring in the vertebrate host, as suggested by several studies [37], or by ecological factors such as the trophic preferences of the vectors [38] or the fact that gorillas’ and chimpanzees’ home ranges might not overlap in space and time. Additional studies would be needed to disentangle these different possibilities.

No human *Plasmodium* species were found. This result is congruent with other studies performed so far on wild populations of apes [8,12,39] thus confirming that, contrarily to what some authors have feared [7,40], great apes do not (and will certainly never) constitute reservoirs of *Plasmodium*, in particular *P. falciparum*, for humans. The fact that their populations are rapidly declining [41,42] is unfortunately another element in support of this prediction. Finally, no evidence of ape-to-human transfers of *Laverania* species was ever recorded despite efforts to find them [37]. The only documented record of this kind of transfer, in a natural context, was for a *P. vivax-*like (non-Laverania) parasite [13].

Regarding the prevalence of infections, more than 15% of the chimpanzee and 21% of the gorilla samples were positive to *Plasmodium*. Infections were detected in 17out of 23sites for chimpanzees and 16 out of 24 for gorillas. Global rates of infection found in this study are similar to those found by Prugnolle *et al.* [2], Kaiser *et al.* [8], and Liu *et al*. [12] in other areas. As previously discussed [2,12], it is very likely that the accurate rates of infection are higher, because the detection of *Plasmodium* in this kind of biological material (faecal) is expected to be less sensitive than in blood, as it is the case for urine and saliva [12,43,44], due to sample degradation or repeated sampling (faecal samples from the same individual may have been collected several times). The effect of sample degradation (and hence DNA degradation) is evident here when comparing the rates of infection detected in the faeces that were collected before and after 24 hours post excretion. The freshest (and so the less degraded) faecal samples significantly present more *Plasmodium* infections than the other ones (odds ratio = 2.038).
